# A Molecular Approach for the Detection and Quantification of *Tribolium castaneum* (Herbst) Infestation in Stored Wheat Flour

**DOI:** 10.17113/ftb.59.01.21.6902

**Published:** 2021-03

**Authors:** Aditi Negi, Arunkumar Anandharaj, Sureshkumar Kalakandan, Meenatchi Rajamani

**Affiliations:** 1 Department of Primary Processing, Storage and Handling, Indian Institute of Food Processing Technology, 613005 Thanjavur, Tamil Nadu, India; 2 Bharathidasan University, 620024 Tiruchirappalli, Tamil Nadu, India; 3 Department of Food Safety and Quality Testing, Indian Institute of Food Processing Technology, 613005 Thanjavur, Tamil Nadu, India

**Keywords:** insect detection, wheat flour, *Tribolium castaneum*, qRT-PCR, red flour beetle

## Abstract

**Research background:**

The presence of insect fragments is one of the major constrains in stored food commodities and it causes considerable loss in the quality of the produce. The management of the pest is viewed as a huge challenge in foodprocessingindustry. Conventionally, the detection of *Tribolium castenaum* in the food processing industry is carried out by acid hydrolysis and staining methods that are time consuming and lack precision.

**Experimental approach:**

Considering the importance of a quick and effective method, a quantitative polymerase chain reaction (qPCR)-based approach was developed and elucidated in this study. The mitochondrial cytochrome oxidase I (*mtCOI*) gene was identified as a target due to its abundance in the pest. Specific primers were designed against the target gene by Primer Premier software and amplified in a qPCR.

**Results and conclusions:**

This method is capable of detecting all the ontogenic stages of *T. castaneum* in stored wheat flour. Earlier experiments had demonstrated that about 20 µg of DNA can be obtained from 2.2 mg of insects. To quantify the infestation levels, the cycle threshold (Ct) values obtained from known samples were subjected to regression analysis and expressed as adult equivalents. In the unknown samples, the infestation was calculated as 1.74 and 0.046 adult insects in 5 g of wheat flour. The maximum permissible limit of insect fragments in flour is 75 insect fragments or approx. 3 adults per 50 g of flour as per the US Food and Drug Administration (FDA). Hence, by adopting this new method, it is possible for the warehouse operators to arrive at a decision to proceed with efficient management practices where wheat flour is stored. Also, this method can be ratified by government agencies associated with international business to ascertain whether the wheat flour meets the standards set by the respective country before subjecting to foreign trade.

**Novelty and scientific contribution:**

This study is the first of its kind in the detection and quantification of *T. castaneum* in milled products. So far, only conventional methods have been employed to assess the presence of the pests and manual counting of fragments are practiced to quantify the infestation levels. The developed qPCR method is faster, reliable and can be employed in milling industries, bakery industries, food processing plants and foreign trade units for critical detection and quantification of *T. castaneum* pest infestation.

## INTRODUCTION

Wheat is one of the major cereal crops cultivated in India and its production has reached an all-time high of 99.70 million tonnes in 2019 (*1*). Globally, India ranks second in the production of wheat, despite the total postharvest losses of about 33.5% in stored wheat and wheat flour (*2*). The major issue is the presence of insects, which is responsible for 5-15% loss during storage (*3*). *Tribolium castaneum* (red flour beetle) is one of the major pests infesting wheat flour during post harvest processing. Even when high standards of sanitary and handling procedures are practiced, the flour is susceptible to infestation by pests (*4*).

The adults of *T. castaneum* are long living, often up to 3 years and undergo complete metamorphosis during their life cycle. The female insect lays up to 300-500 eggs during her life span. Complete life cycle of the insect ranges from 7 to 12 weeks depending on temperature (35 ºC) and relative humidity (60-80%) (*5*). Larva and adults are the active feeding stages of *T. castaneum* and cause significant damage to food commodities. During postharvest handling and storage, the grain seeds are often broken or wounded by other internal feeders, which makes it easier for the red flour beetle to infest them (*6*). The pest infestation causes severe damage to the products, like contamination with faeces (mainly uric acid), which increases the humidity of the flour, promoting hot spots for fungal or mould growth. Besides the contamination with the body fragments and frass, the flour is also exposed to quinones that are secreted by the adult insects as a defence mechanism (*7*). These quinones are responsible for the unpleasant odour and are known to induce liver and spleen tumours, when tested in rats and other vertebrates (*8*, *9*). The quinones also affect humans by causing several complications like jaundice, anaemia, allergic responses, *etc*. The product quality and marketability of the flour is greatly reduced due to the infestation, resulting in the huge economic loss.

In the food industry, the quality and integrity are considered as important criteria for the successful export/import of any produce in the market. The insects present in the broken wheat are crushed during milling and thereby the fragments are blended to the flour and ultimately affect the quality of the flour. In European countries, there is no systematic surveillance or any scientific risk assessment programs on storage of pests, *albeit* a zero or nil tolerance for insect or insect fragments has been established in food and food products (*10*, *11*). In the USA, the Food and Drug Administration (FDA) has a defect action level of 75 insect fragments per 50 g of wheat flour (*12*), whereas in Canada the defect action level should not exceed more than 25 fragments per 50 g of flour (*13*).

Currently, various techniques employed to detect insects and their fragments in stored food commodities are based on: (*i*) sieving, (*ii*) physical examination, (*iii*) microscopic methods such as staining and acid hydrolysis, (iv) enzyme-linked immunosorbent assay (ELISA) (*14*, *15*), (*v*) near infrared spectroscopy (NIR) (*16*), (*vi*) filth flotation (*17*), and (*vii*) soft X-rays (*18*-*22*). Although some of these methods are effective for detecting a particular stage of *T. castaneum* during infestation, yet none of these can detect all the developmental stages of the insect. Notably, detection of *T. castaneum* at initial stages of its life cycle is difficult. Due to stringent regulations followed by the government during import and export, it is imperative that the flour should be devoid of any insect or insect fragments before it is allowed to foreign trade. Hence, there is an urgent need to develop a new method to detect and quantify *T. castaneum* infestation at all ontogenic stages present in wheat flour. To the best of our knowledge, no study has been conducted utilizing qPCR for identifying insect infestation in stored wheat flour. This is the first study of its kind to use a qPCR technique that specifically amplifies a portion of *mtCOI* (mitochondrial cytochrome oxidase) gene to successfully check and assess *T. castaneum* infestation in stored flour.

## MATERIALS AND METHODS

### Insect specimen collection and rearing

Major stored grain pests, namely *Tribolium castaneum*, *Sitophilus oryzae* and *Lasioderma serricorne* cultures, were maintained at Indian Institute of Food Processing Technology (IIFPT), Primary Processing, Storage and Handling Laboratory, Thanjavur, India. The cultures of *S. oryzae* were maintained on whole wheat and *T. castaneum* and *L. serricorne* in wheat flour at 30 ºC and 70% relative humidity (RH). All stages of the insect development, *viz.* egg, larva, pupa and adults were maintained separately for the experimental study.

### Staining of T. castaneum eggs and fragments

The *T. castaneum* eggs were stained with iodine as per the standard procedure described in AACC method 28-44.01 (*23*). About 50 g of *T. castaneum-*infested wheat flour was mixed with 500 mL of 5% HCl (Hi-Media, Nashik, India) and light mineral oil. The sample was boiled for 10 min with constant stirring to ensure complete digestion. The digested sample was transferred to a separating funnel and allowed to stand at room temperature until phase separation was clearly visible. The lower layer was drained off leaving about 2.5 cm of the interface. The sample was then washed three times with hot water, followed by phase separation in a separating funnel. After the final wash, the sample was filtered through a lined filter paper and examined according to AACC method 28-41.03 (*24*) under a microscope (Leica StereoZoom S8 APO with LAS (Leica Application Suite) software v. 3.8.4; Leica, Singapore). The insect fragments present were counted and photographed.

### Insect DNA extraction procedures

About 30 mg of *T. castaneum* (egg, larva, pupa and adults) and adults of  *L. serricorne* and *S. oryzae* were ground in liquid nitrogen using a clean glass rod to make a fine powder. DNA from egg, larva, pupa and adults was isolated using HiPurA™ (insect DNA purification kit MB529; Hi-Media) as per manufacturer’s instructions. Finally, the total DNA was eluted in 200 µL of elution buffer (10 mM Tris-Cl, pH=8.5) by brief centrifugation (Centric 200R; Tehtnica, Železniki, Slovenia) at 16 128×*g*. Efficiency and integrity of the isolated DNA was checked by agarose gel electrophoresis and the purity was analyzed spectrophotometrically (UV 1800; Shimadzu, Sapporo, Japan) by measuring the absorbance at _260 nm_ and _280 nm_. The extracted DNA was stored at –20 ºC until use for experiments.

### Wheat DNA isolation

Wheat grains purchased from the market were screened to ensure that there was no visible infestation. About 5 g of wheat grains were finely ground with a mortar and pestle and the powder was suspended in extraction buffer (100 mM Tris(hydroxymethyl) aminomethane (Tris)-HCl (pH=8.0), 50 mM EDTA (pH=8.0), 500 mM NaCl, 1% sodium dodecyl sulphate (*m*/*V*); Hi-Media) and vortexed for a few minutes. The contents were allowed to settle and 3 M potassium acetate at pH=5.2 (Hi-Media) was added, then the whole solution was vortexed and centrifuged (Centric 200R; Tehtnica) at 15 700×*g* for 10 min. The upper layer was aspirated and approx. 350 µL of prechilled isopropanol were added to the sample, which was then incubated at -20 °C. The pelleted DNA was centrifuged and the supernatant was discarded, followed by washing of the pellet with ethanol twice and air dried. The pellet was dissolved in 100 µL of Tris and EDTA (TE) buffer and analyzed by agarose gel electrophoresis (Bio-Rad, Irvine, CA, USA) for consistency.

### Designing the primer sequence and specificity analysis

Specific primers were designed using Primer Premier 6 software available online (*25*) to target *T. castaneum* mitochondrial cytochrome oxidase I (*mtCOI*) gene (GenBank (*26*) accession number EU048277.1) and synthesised by Eurofins Genomics India Pvt. Ltd., Bangalore, India. Multiple sequence alignment was performed using Gene Runner program v. 6.5.52 Beta (*27*) and a comparison of *mtCOI* gene among *T. castaneum*, *L. serricorne* and *S. oryzae* was done to ensure the designed primers does not overlap with genes of other storage pests.

### Internal control/reference gene

The Ct values of the target gene were normalized with Ct values of an internal control or reference gene. Here we used *T. castaneum* glyceraldehyde 3-phosphate dehydrogenase (GAPDH) gene, GenBank (*26*) accession number XM_969088, as a reference for this study. The primer sequence for GAPDH gene used in the study is 5'-GGACGCCTACGACCCTTCAG-3' and 5'-GTCATCAACCCCTCCACAATCT-3’ for leading and lagging strands respectively (*28*) and the primer sequences were synthesised by Eurofins Genomics India Pvt. Ltd., Bangalore, India.

### PCR reaction

PCR amplification (Veriti™ 96-well thermal cycler; Applied Biosystems, Foster City, CA, USA) was performed in a reaction volume of 20 μL, containing 10 μL of 2X Master Mix (Aura, Chennai, India), 0.2 μM of forward and reverse primers and the 100 ng/reaction of template DNA (*29*, *30*). The PCR conditions used for the amplification were as follows: initial denaturation at 95 ºC for 10 min, followed by 40 cycles consisting of denaturation at 95 ºC for 30 s, annealing at 60 ºC for 30 s, extension at 72 ºC for 30 s and final extension at 72 ºC for 5 min. The PCR products including non-target controls were electrophoresed together on a 1.5% agarose gel along with 1-kb ladder. The gel was stained with ethidium bromide and the bands were visualized under UV light.

### Quantitative real time polymerase chain reaction (qRT-PCR)

DNA samples isolated from *T. castaneum, L. serricorne* and *S. oryzae* were amplified using thermal cycler (LightCycler 96; Roche, Penzberg, Germany). qPCR reaction mixture (20 µL) contained 10 µL fast start essential SYBR Green PCR Master Mix (Roche Diagnostics, GmbH, Indianapolis, IN, USA), 2 µL (0.2µM) of each *T. castaneum* specific forward and reverse primers, and 5 µL of DNA corresponding to 100 ng/reaction served as a template for the study. The amplification conditions were followed as described by Amin *et al.* (*31*). Samples without template DNA were used as negative control and the DNA isolated from the adult red flour beetle was used as a positive control.

### Regression analysis for quantification of T. castaneum

About 5 g of wheat flour was mixed with DNA isolated from adult *T. castaneum* at a concentration of 200, 100, 50, 25, 12.5, 1.25 and 0.125 ng, which was equivalent to 10, 5, 2.5, 1, 0.1, 0.01 and 0.001 adult insects respectively. The total DNA was then isolated from the infested flour and amplified by qPCR as mentioned previously. Regression analysis was performed for the Ct values obtained from the aforementioned seven different concentrations, and it was repeated six times as individual experiments, obtaining altogether 42 samples. All the experiments were run along with the DNA either from *L. serricorne* or *S. oryzae,* which served as two negative controls in addition to only wheat DNA.

### Analysis of defect action level of insect fragments in wheat flour

To examine the possibility of application of the developed method in unknown samples, wheat flour was purchased randomly from a commercial store (two samples from a popular brand, *viz.* Aashirvad, Chennai, India and Naga, Dindigul, India) and a local mill (three samples) located in the city of Thanjavur. The DNA extracted from the wheat flour was subjected to qRT-PCR analysis as described earlier.

### Data analysis

Statistical analysis was performed using GraphPad Prism program, v. 8 (*32*) and two-tailed *t*-test was carried out for all stages of *T. castaneum* (egg, larva, pupa and adult). ANOVA served to compare among the groups and values p<0.05 were considered statistically significant. The Ct values obtained from qRT-PCR were inversely proportional to the quantity of original template DNA. In order to obtain a regression for the quantification of insect infestation in wheat flour, a regression analysis was performed at 95% confidence level.

## RESULTS AND DISCUSSION

### Primer specificity and multiple sequence alignment

**Fig. 1:**
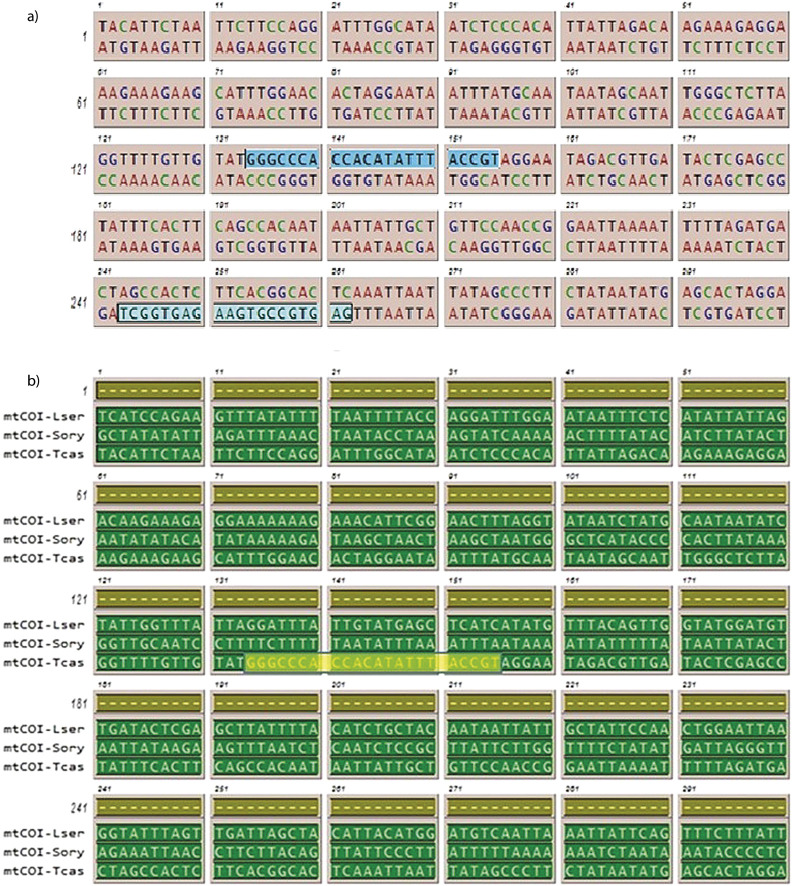
Multiple sequence alignment of mitochondrial cytochrome oxidase I (*mtCOI*) gene: a) the gene sequence of *Tribolium castaneum mtCOI* gene; highlighted is the region of primer binding, and b) alignment of *mtCOI* sequence from *Tribolium castaneum, Lasioderma serricorne* and *Sitophilus oryzae* available in GenBank (*26*) under accession numbers: EU048277.1, KU494128.1, and NC_030765.1 respectively. The alignment was done by Gene Runner program (*27*), which shows no similarity in the same gene of other storage pests; highlighted is the forward primer-binding region specific to *T. castaneum*

The main aim of this study was to develop a molecular technique to detect and quantify the presence of red flour beetle in wheat flour. qRT-PCR is a well-known technique based on gene expression for rapid detection and quantification of *T. castaneum* in food samples. The *mtCOI* gene of *T. castaneum* is used as a target, due to its abundance and availability in the cell as multiple copies (*33*), thus greater sensitivity can be achieved if it was used as a candidate for identifying infestation. The primer for *mtCOI* gene was designed using Primer Premiere application with options set in the primer probe design and multiplex PCR mode. Several forward and reverse primers were generated from the program that were sorted for suitability based on the recognition site, the annealing site corresponding to the template sequence, the *t*_m_ value (melting temperature), guanine-cytosine (GC) ratio, no primer-dimer formation (self-annealing and self-complementarity) and no possibility of hairpin-loop formation. The primer sequence is 5'-GGGCCCACCACATATTTACCGT-3' complementary to leading strand to serve as forward primer and 5'-GAGTGCCGTGAAGAGTGGCT-3’ complementary to lagging strand to serve as reverse primer (Fig. 1a).

**Fig. S1:**
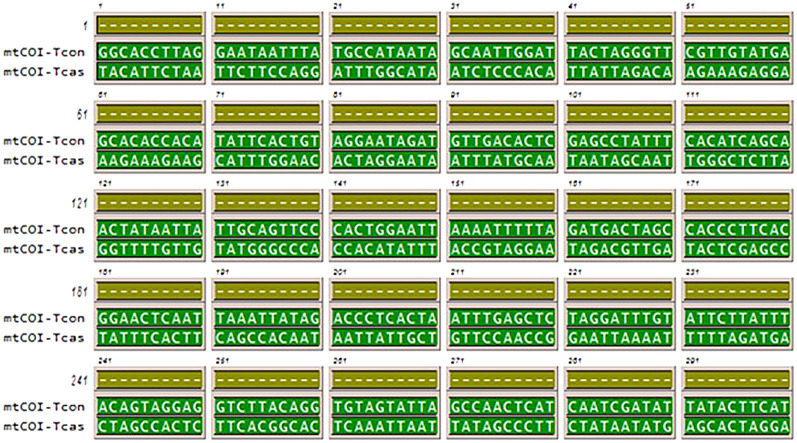
Multiple sequence alignment between closely related species of *Tribolium castaneum* and *T. confusum* shows that there are no species similarities exist in themitochondrial cytochrome oxidase I (*mtCOI*) gene

As the stored wheat flour is prone to harbouring many pests, it is important to ascertain that the designed primer does not cross-react with *mtCOI* gene of other stored pests. Hence, a multiple sequence alignment of *mtCOI* gene from all three major stored pest was carried out, which indicated that the red flour beetle gene shares 75 and 43.4% homology with *S. oryzae* (internal feeder) and *L. serricorne* (external feeder) respectively. It was observed that *T. castaneum* primers could not anneal to *mtCOI* gene (Fig. 1b) of other major pests of wheat flour. Additionally, to reaffirm that the primers do not cross-react with closely related species within the same genus, multiple sequence alignment comparison was made between *T. castaneum* and *T. confusum.* The results indicated that the primer is specific only to *T. castaneum* (Fig. S1) and thus it is proved that *mtCOI* gene is the ideal candidate for the present study. Elbrecht *et al.* (*34*) reported that ideal primer sets with optimal annealing temperatures play a significant role in minimizing the amplification bias among the taxa to maximize species recovery.

### Primer specificity and sensitivity

**Fig. 2:**
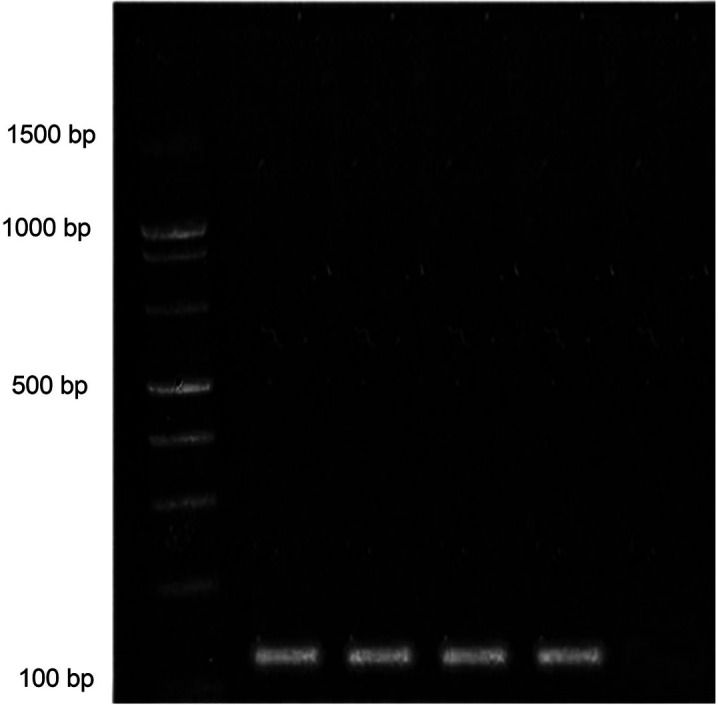
Electrophoresis of the polymerase chain reaction (PCR) products on 1.5% agarose gel shows amplification product (128 bp) using primermitochondrial cytochrome oxidase I(mtCOI). Lane1=1.5-kb ladder, lane 2=*Tribolium castaneum* adults, lane 3=*T. castaneum* pupa, lane 4=*T. castaneum* larva, lane 5=*T. castaneum* egg, lane 6=*L. serricorne* adult as negative control

**Fig. S2:**
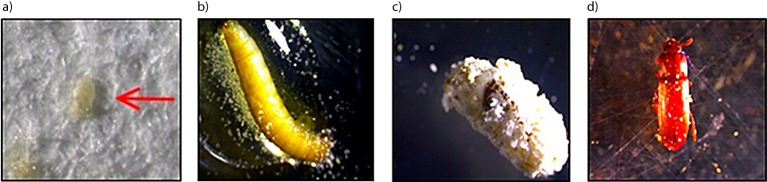
Staining by iodine method of *Tribolium castaneum*: a) egg, b) larva, c) pupa, and d) adult

**Fig. S3:**
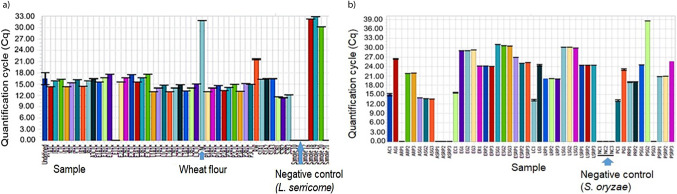
Quantitative real-time polymerase chain reaction (qRT-PCR)-based amplification showing no amplification in negative control: a) *Lasioderma serricorne* and b) *Sitophilus oryzae*

**Fig. S4:**
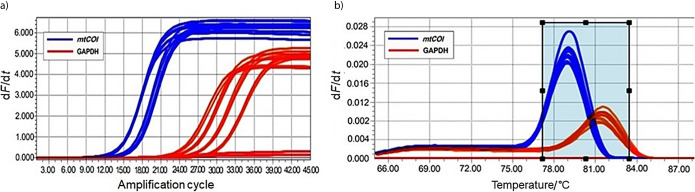
Quantitative real-time polymerase chain reaction (qRT-PCR) analysis of *Tribolium castaneum* with mitochondrial cytochrome oxidase I (*mtCOI*) and GAPDH primers: a) analysis of the qPCR with *mtCOI* primer, glyceraldehyde 3-phosphate dehydrogenase (GAPDH) served as an internal control for normalization of data with all stages of *T. castaneum* (egg, larva, pupa and adult). Adults of *Lasioderma serricorne* were used as a negative control. All positive reactions amplified in the logarithmic phase before 29 cycles for GAPDH gene (dark orange) and 16 cycles for *mtCOI* gene (blue). All reactions show single melting peak and no amplification was observed for negative control, and b) melting curve analysis of *T. castaneum* DNA with *mtCOI* and GAPDH primers shows positive single peak obtained for *T. castaneum* with both primers. (-d*F*/d*t*)=negative derivative of fluorescence over temperature

To analyse the sensitivity of the primer and its ability to detect *T. castaneum* at all ontogenic stages, DNA was purified from egg, larva, pupa and adults that were subjected to PCR amplification (Fig. S2). The results indicated that the sensitivity of the primer is high, that amplification is observed in the DNA samples purified from different ontogenic stages of *T. castaneum*, while no amplification is noticed in the DNA sample from *L. serricorne* (Fig. 2 and Fig. S3). To increase the stringency level of detection and to avoid false positives, several qRT-PCR assays were performed from the DNA isolated from different sets of samples. The obtained Ct values were tight and a single peak melting curve was observed after every run. In our study, samples with Ct value equal to or above 30, for internal control or samples, were considered negative. The amplification curve appeared in the12th cycle for pupal stage, in the 15th cycle for egg stage, and the Ct values for larva and adult were in between these two stages (Table 1 and Fig. S4a). The obtained amplification curves appeared as a single bundle, indicating that there was no off-target amplification at any growth stage of *T. castaneum* along the whole run. Melting curve analysis also showed specific melting temperature (*t*_m_) of (78.96±0.09) °C for all *mtCOI* amplification products of *T. castaneum* and (81.4±0.1) °C for GAPDH (Fig. S4b). A single sharp peak was observed in the melting curve of control sample and there were no non-specific products and primer dimer observed in the qPCR reaction. Similarly, no amplification or other non-target amplifications were detected in the negative sample, which indicates the specificity of the designed primer used in the study. Previous studies on qPCR data analysis revealed that a single melting curve ensures that the detected signal is genuinely the target of interest (*35*).

**Table 1:** Mean cycle threshold (Ct) values of all ontogenic stages of *Tribolium castaneum*

*T. castaneum* growth stage	Ct value	
GAPDH	*mtCOI*
Egg	29.03±0.03	(15.63±0.06)^a^
Larva	24.34±0.15	(13.12±0.26)^b^
Pupa	22.97±0.23	(12.96±0.29)^c^
Adult	26.37±0.17	14.99±0.33

Different letters indicate significant differences between the adult and other growth stages at: ^a^p<0.01, ^b^p<0.05, and ^c^p<0.01. Each value is represented as mean±S.D. (*N*=9). GAPDH=glyceraldehyde 3-phosphate dehydrogenase, *mtCOI*=mitochondrial cytochrome oxidase I

### Quantification of T. castaneum infestation by regression analysis

**Fig. 3:**
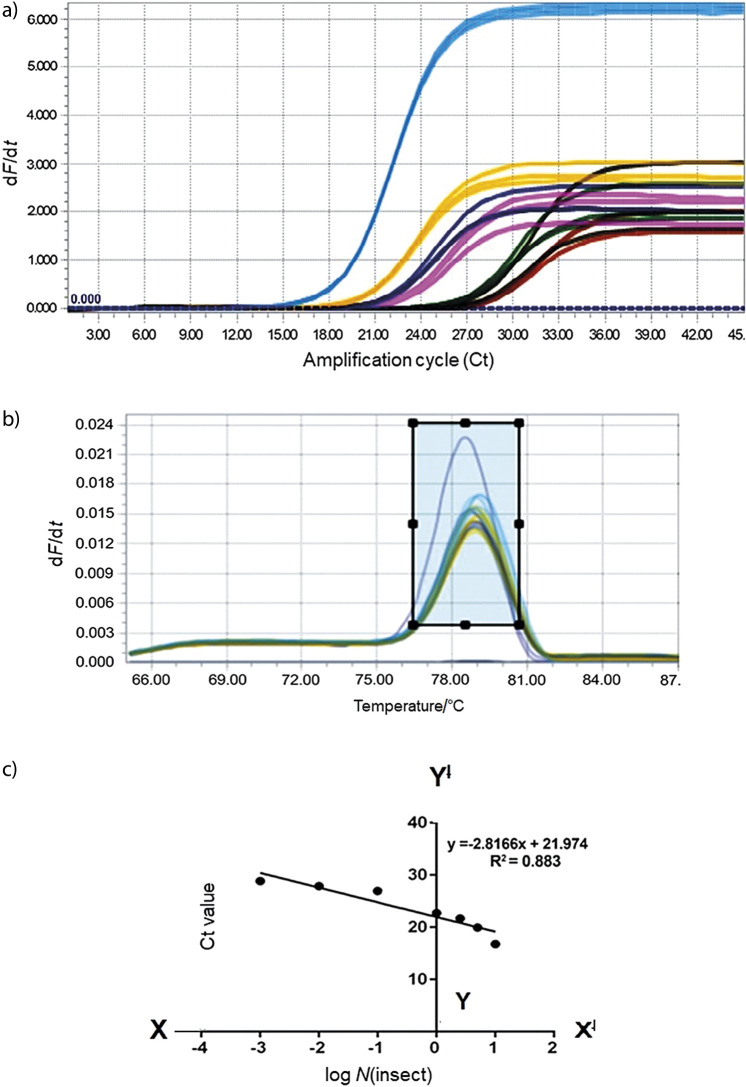
Quantification of *Tribolium castaneum* infestation by regression analysis and authenticity of *T. castaneum* detection in infested wheat flour: a) amplification curves of *T. castaneum* in quantitative polymerase chain reaction (qPCR) reaction. Decreasing mass of *T. castaneum* adult DNA (ng) from 200, 100, 50, 25, 12.5, 1.25 to 0.125 by serial dilutions with wheat DNA to emulate number of insects 10, 5, 2.5, 1, 0.1, 0.01, 0.001 in 5 g of wheat flour. All the insect samples entered logarithmic phase before 29 cycles of reaction, while the wheat DNA used as negative control entered after 33 cycles of amplification, b) melting curve analysis of all serially diluted *T. castaneum* DNA shows positive single peak for *T. castaneum mtCOI* at (78.96±0.09) °C, and c) regression plot for  *T. castaneum* adult DNA shows significant correlation between the log number of insects and cycles threshold (Ct) values in 5 g of wheat flour (p<0.0016 and R^2^=0.883). (-d*F*/d*t*)=negative derivative of fluorescence over temperature.

Serial dilutions of the DNA from adult *T. castaneum* ranging from 200 to 0.125 ng were mixed with 5 g of wheat flour. We used 0.125 ng of DNA as the minimum detection level, below which the Ct values were above 30, which is considered undetectable or negative, and further loss of linearity was also observed (Table 2, Fig. 3a and Fig. 3b). To increase the reproducibility of the obtained results in each qRT-PCR run, 200 ng of adult *T. castaneum* DNA was added as positive control, for which the Ct value was around 16.8±0.02.

**Table 2:** Cycle threshold (Ct) values obtained for *Tribolium castaneum* in 5 g of wheat flour used for regression analysis

Sample	*m*(DNA)/ng	*N*(insect equivalent)	Ct value
*T. castaneum* adult DNA diluted with wheat DNA	200	10	(16.8±0.02)^a^
	100	5	(20.0±0.1)^a^
	50	2.5	(21.67±0.09)^a^
	25	1	(22.7±0.2)^a^
	12.5	0.1	(27.0±0.2)^a^
	1.25	0.01	(27.9±0.3)^a^
	0.125	0.001	(28.84±0.08)^a^
Wheat DNA	108	NA	34.4±1.2

^a^Significant difference between all *Tribolium* DNA mass and wheat DNA at p<0.01 level of significance. Each value is represented as mean±S.D. (*N*=3). NA=not available

As *T. castaneum* may be detected in the DNA isolated from infested wheat flour, the exact quantity of the insect DNA present in the total DNA might vary with samples. Our data indicated that a minute quantity of insect DNA is sufficient to observe the amplification. The observed Ct values were so tight (±1 Ct) for each stage of the insect, which signifies the conformity of the developed method. Statistical analysis of inter-stage comparison showed a significant change in the expression of *mtCOI* gene between all stages of the insect growth except for larva and pupa. We presume that there may be fewer developmental changes during the larval and pupal stages, which may be a factor that no significant change in the expression of *mtCOI* (*36*) was observed.

A standard curve was generated by using different mass of *T. castaneum* DNA for quantitative analysis in the wheat flour. Regression analysis revealed statistically significant relation between the DNA mass (mass/infestation dose) and the obtained Ct values (p<0.0016 and R^2^=0.883) (Fig. 3c). There was an inverse correlation (described below by the equation for a slope) between the infestation and the Ct values: as the rate of infestation decreased, the Ct values increased:

y=mx+c /1/

where y stands for Ct value from adult insect, x represents the insect infestation dose in wheat flour, and c is a constant. The details of the statistical analysis are presented in Table S1.

y=2.817x+21.97 /2/

R^2^=0.883

Here R^2^ is a goodness-of-fit measure for linear regression models. The regression line was plotted using Ct values, which transform correspondingly into the adult equivalents by using Eq. 2. It is understood that the mass of DNA directly correlates with the biomass of the insect, so this technique can be used for quantifying the infestation level present in wheat flour. As reported earlier, the mean fresh mass of adult *T. castaneum* is about (2.40±0.03) mg, of larva and pupa (2.30±0.06) mg and egg about (0.0052±0.0008) mg (*37*). Our data indicate that 20 µg of DNA directly corresponds to 2.2 mg fresh body mass of *T. castaneum*. Based on the generated regression curve, the Ct values approached 17, which represents approx. 10 adults present in 5 g wheat flour. Similarly, the Ct value near 28 represents 0.001 adult (Table S2). Thus, the inverse prediction of the Ct values will help in detection and quantification of *T. castaneum* infestation in stored wheat flour, and help in most of the management decisions to safeguard the product. By using a regression curve, it is possible to determine the minimal defect action level of insect fragments present in unknown samples. Our studies are in line with the results reported for *Rhyzopertha dominica* infestation in rice (*38*).

Other confounding factors such as high gluten content and polysaccharides present in the wheat flour pose an impediment in the amplification of DNA by interfering with the activity of Taq polymerase in PCR reaction (*39*, *40*). Our results suggested that these factors did not significantly affect the qRT-PCR assay. The smallest level of infestation that can be detected by our method is 1 insect fragment in 5 kg of flour, which is much lower than the permissible level allowed in countries like the USA and Canada as per the recommendation for minimum defect action level by FDA and CFIA (*10*).

### Authenticity of T. castaneum detection in the infested wheat flour

**Fig. 4:**
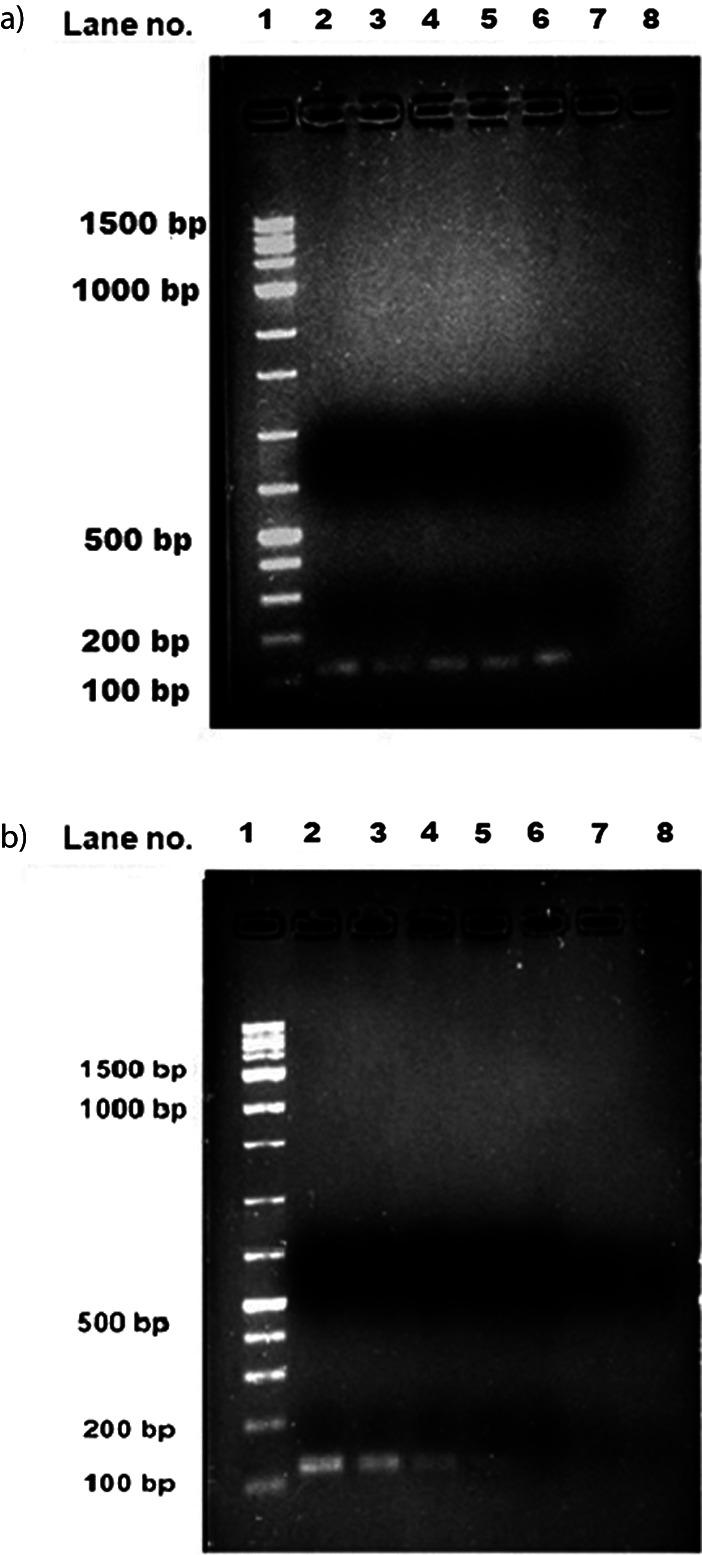
The results of quantitative polymerase chain reaction (qPCR) analysis show: a) a sequential increase in the amount of amplifiedmitochondrial cytochrome oxidase I (*mtCOI*) gene corresponding to the mass of insect DNA present in wheat flour. Lane 1=1.5-kb ladder, lane 2=amplified product from *T. castaneum* adult DNA (positive control), lane 3=wheat flour with one insect fragment, lane 4=wheat flour with two insect fragments, lane 5=wheat flour with five insect fragments, lane 6=wheat flour with ten insect fragments, and lane 7=*S. oryzae* adult DNA as negative control, and b) DNA isolated from 5 g of wheat flour purchased from a commercial store and amplified with *mtCOI* primer. The qPCR products loaded in lane1=1-kb ladder, lane 2=amplification product from *T. castaneum* DNA (positive control), lane 3=local flour mill sample 1, lane 4=local flour mill sample 2, lane 5=local flour mill sample 3, lane 6=commercial store flour sample 4, lane 7=commercial store flour sample 5, lane 7=*S. oryzae* adult DNA as negative control

**Fig. S5:**
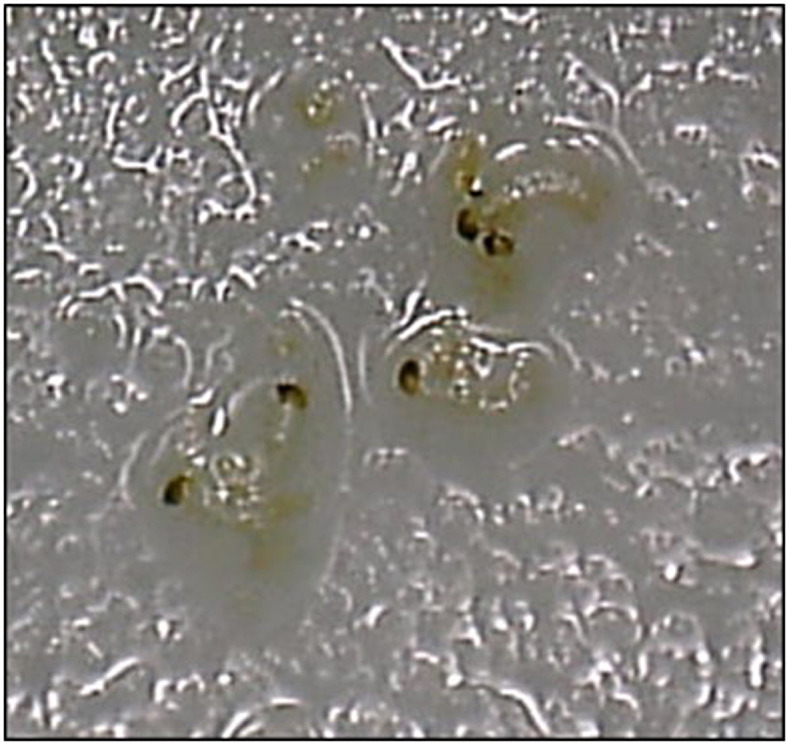
*Tribolium castaneum* fragment identification in wheat flour by acid hydrolysis method

To determine the reliability of the developed method, a known number of fragments from  *T. castaneum* were blended with a certain amount of wheat flour. The presence of fragments in the flour was confirmed by staining according to the protocol described in AACC Method 28-41.03 (*24*) (Fig. S5). Then, whole DNA was extracted from the flour containing the fragments and subjected to qRT-PCR analysis. The results indicated that the developed method is sensitive enough to detect the presence of insect infestation at the level of one fragment in 5 g of wheat flour and the Ct value was (29.03±0.04). The number of fragments present in the wheat flour is inversely proportional to the Ct values obtained from the experiment. In our experiment the Ct values 27.32, 24.27 and 13.25 indicated the presence of 2, 5 and 10 insect fragments in 5 g of wheat flour respectively. Our study clearly showed that this method is highly sensitive and able to detect one insect fragment in 5 g of wheat flour (Table 3 and Fig. 4a).

**Table 3:** Cycle threshold (Ct) values for different *Tribolium castaneum* threshold samples

Sample	Ct value
*T. castaneum* Std. (adult DNA)	18.4±0.1
T1 (1 insect fragment)	29.03±0.04
T2 (2 insect fragments)	27.3±0.4
T3 (5 insect fragments)	24.3±0.3
T4 (10 insect fragments)	13.2±0.4

Each value is represented as mean±SD (*N*=3)

The developed method was challenged by testing five different blind samples, which were purchased from a local mill and a commercial store. The samples procured from the local mill showed positive results for the presence of insect fragment and the Ct values were 22.6, 25.7 and 32.3 (Fig. 4b and Table 4), whereas the Ct values 30.7 and 30.63 were observed for samples belonging to the popular brand. Correspondingly, the Ct values were translated to infestation levels by substitution in Eq. 2. The infestation level was 1.74 (sample S1) and 0.046 (sample S2) adults per 5 g of wheat flour, while the rest of the samples exhibited negligible amounts. The amplified product (128 bp) from qPCR run was confirmed by agarose gel electrophoresis. The method demonstrated in this study can be adopted for the identification and/or quantification of *T. castaneum* infestation in different grains and types of flour such as almond, amaranth, barley, buckwheat, cassava, corn, garbanzo, millet, oat, potato, quinoa, rice, rye, sorghum, spelt and coconut (*41*). Thus, based on our method, it is possible to differentiate between infested and non-infested samples with high sensitivity.

**Table 4:** Cycle threshold (Ct) values for quantitative real-time polymerase chain reaction (qRT-PCR) with primer specific for *Tribolium castaneum* in the samples obtained from a local mill and a commercial store

Sample	Ct value	*N*(insects as adult equivalent) per *m*(flour)=5 g
S1	22.6±0.2	1.74
S2	25.7±0.0	0.046
S3	32.3±0.3	0.0002
S4	30.7±0.2	0.0007
S5	30.63±0.06	0.0007

Each value is represented as mean±S.D., *N*=3, S1-S3=samples of wheat flour from local mills, S4 and S5=samples of wheat flour from local stores

## CONCLUSIONS

The developed qRT-PCR method is highly accurate and species specific that it detects only *Tribolium castaneum* infestation in a given sample. There were no false positive results obtained when tested against a closely related species of the same genus, *i.e. T. confusum.* Similar results were also obtained when tested with other stored pests such as *Sitophilus oryzae and Lasioderma serricorne*. To the best of our knowledge, this is the first study to quantify *T. castaneum* infestation in stored wheat flour using qRT-PCR. Since the method is a rapid technique, it can be adapted in warehouses to effectively manage the pest infestation. Furthermore, governmental agencies responsible for import and export of commodities like wheat flour can use this method to determine if the commodity has infestation below the defect action level. Small sample size will help to analyze more samples from a single batch of stored flour.

## Figures and Tables

**Table S1:** Data obtained from three independent experiments using regression analysis. Correlation was obtained for the cycle threshold (Ct) value with the corresponding infestation dose. The final equation derived from the analysis was used for calculating infestation in the flour in adult equivalents

Parameter for regression analysis	Obtained best-fit value for regression analysis
Slope	-2.8±0.4
y-intercept	21.9±0.68
x-intercept	7.8
1/Slope	-0.355
Confidence interval	95%
Slope	-3.990 to -1.643
y-intercept	20.22 to 23.73
x-intercept	5.295 to 13.82
Goodness-of-fit	
R^2^	0.8839
Sy.x	1.675
F value	38.06
Degree of freedom for numerator and denominator	1,5
p-value	0.0016
Deviation from horizontal axis	Significant
Data	
Number of xy pairs	7
Equation	y=-2.82·x+ 21.97

**Table S2:** Frequency of DNA detection at different insect densities present in stored wheat flour

Insect equivalents as adults	*N*(*T. castaneum* beetle)/(*m*(wheat flour)/g)	Ct value
10	10/5	16.8±.02
1	1/5	22.73±0.23
0.1	1/50	26.95±0.17
0.01	Corresponds to 1/500	27.87±0.32
0.001	Corresponds to 1/5000	28.84±0.08

Each value is represented as mean±S.D. (*N*=7), Ct=cycle threshold
